# Improvement of motor disorders and autistic symptomatology by an approach centered on the body axis: a two-case report

**DOI:** 10.3389/frcha.2025.1451559

**Published:** 2025-04-14

**Authors:** Sylvie Pussino, François Darchen

**Affiliations:** Centre d’Action Médico-Sociale Précoce, Gonesse Hospital, Gonesse, France

**Keywords:** autism spectrum disorder, motor impairment, body axis, psychomotricity, case report, motor impairments

## Abstract

Motor dysfunction is commonly associated with autism spectrum disorders (ASD). However, even if it may represent an intrinsic dimension of ASD it is not thought of as a relevant therapeutic target. Here we describe the postural, motor, and autistic characteristics of two children with ASD, a girl aged 3 years and 9 months and a boy aged 4 years and 7 months at enrollment, and their evolution over 20 or 23 months in response to interventions targeting these postural characteristics. Both met DSM-5 diagnosis critera for ASD. In both cases, asymmetric postures, twisting around the longitudinal axis, and underuse of the hands, particularly the thumbs, were observed. The children were repeatedly encouraged to engage in motor experiences involving their spatial cues and body axes and to correct their postures. We assessed more than fifty items describing motor particularities, communication, and social interactions. We observed a progressive and synchronous improvement in most of the items. In particular, communication and interaction skills improved in a similar way to motor skills. CARS scores also improved from 36 at initial assessment to 26 at the end of the follow-up for the boy and from 39.5 to 30 for the girl. These results suggest that motor dysfunction is an intrinsic dimension of autism and that interventions aimed at improving motor organization around a “body axis” could benefit children with ASD.

## Introduction

Autism spectrum disorders (ASD) are characterized by deficits in communication and social interaction, restricted interests and repetitive behavior. Sensory impairments, but not motor dysfunction, are now recognized as diagnostic criteria for ASD (1). However, more than 80% of individuals with ASD have motor coordination impairments, a postural control defect (2–6), gross or fine motor abnormalities (7, 8). Recent reviews have suggested that motor impairments are so prevalent in ASD that they may represent a cardinal feature of ASD (9–13). Accordingly, motor function disorders occur at an early age in ASD (14–21); moreover, there is a correlation between spontaneous movement abnormalities at 4 months of age and an elevated risk of ASD at 18 months of age (22) and a correlation between motor disorders, repetitive behaviors and verbal communication deficits (23–26). The data suggest that the motor disorders observed in ASD relate to disturbances in sensory integration or a failure in the interface between sensory information and action planning.

However, there is a lack of data on the impact of interventions specifically targeting motor function disorders on autistic symptoms.

In our experience, young children with ASD have motor and postural characteristics that suggest the existence of deficits in the organisation of motor skills around the body's axes (longitudinal, sagittal, frontal) and a lack of representation of the body and its movements in space. The lack of intra- and extra-bodily spatial reference points could have a major impact on the development of manipulative skills, the exploration of cause-and-effect relationships and interpersonal relations. To test this hypothesis, we developed a therapeutic approach focusing on the acquisition of harmonious motor patterns around the three axes of the body.

Our first aim was to provide a detailed description of postural and motor characteristics that we found to be associated with ASD. Our second aim was to describe the “body axis-oriented” psychomotricity approach developped in our center to treat children with ASD and to provide evidence for its validity. To do so, we enrolled two children with ASD scored about 50 items to assess motoric and postural peculiarities as well as common autistic symptoms and their evolution over 20 or 23 months.

## Methods

### Patients

Two children with ASD referred to our pediatric center were enrolled. The diagnosis of ASD was informed by clinical observations, ADI-R (27) and Childhood Autism Rating Scale (CARS) (28); it fullfilled DSM-5 criteria (1). The childrens' care consisted of two weekly psychomotor therapy sessions and one weekly psychotherapy session. The local Ethics committee concluded that his approval was not required, as the study was based on the observation of two children receiving psychomotor care integrated in the follow-up of all patients with ASD in our Institution. Written informed consent was obtained from the minors' legal guardians for the publication of any potentially identifiable images or data included in this article.

Saral (first names were changed) was of Indian origin. He was 4 years 7 months old at enrollment. Clinical presentation is provided in Supplementary Methods. According to DSM-5, Saral had level 3 autism for social communication and level 2 for restricted/repetitive behaviors. A score of 34 (i.e., moderately autistic) was obtained with CARS at initial assessment. The following developmental ages were found (in months) with PEP-3 (29): cognition, 28; expressive langage, less than 12; receptive langage, 16; fine motricity, 30; global motricity 24. There was no neurological defect nor comorbidity. Cerebral MRI, EEG, chromosomal analysis on a DNA chip and sequencing of 203 genes associated with intellectual deficiency including FMR1, revealed no abnormalities except a missense variant of unknown significance in *TRIP12* that was also carried by the asymptomatic mother and thus probably not responsible for the phenotype.

Bahiya was of Nigerian origin and the youngest of three siblings. Parents were unrelated, had poor French language skills and spoke english at home. Bahiya's clinical presentation is provided as Supplementary Methods. At the beginning of the study, she was 3 years and 9 months old. According to DSM-5, Bahiya had level 3 autism for social communication and level 2 for restricted/repetitive behaviors. A score of 39.5 (i.e., severely autistic) was obtained with CARS at initial assessment.

She was developmentally delayed as evidenced by her PEP-3 scores (in months) at 45 months of age: cognition, 15; expressive langage, 13; receptive langage, 12; fine motricity, 22; global motricity 22.

There was no comorbidity. Neurological examination, cerebral MRI, EEG, and brainstem auditory evoked potentials revealed no abnormalities. *FMR1* gene sequencing revealed the presence of a premutation with 57 CGG repeats on one allele and 31 on the other, unable to account for the observed phenotype.

### Psychomotricity sessions

The therapist guided the child to develop symmetrical posture, harmonious movements in all three axes and proprioception. Ritualized activities such as taking off the shoes and rhymes at the beginning of the session and putting on the shoes at the end set time limits for the sessions. Some of the activities commonly offered to children are described in Supplementary Methods.

## Results

For both patients, we assessed 50–65 items related to motor impairments or autistic symptomatology over approximately 2 years. For each four-month period, we assigned an “average” score to each item on a scale from 0 to 5, with 5 representing maximum severity (see Supplementary Methods and Supplementary Figure 1). The scores are shown in Tables 1, 2 and Figure 2. A detailed description of these observations is provided as Supplementary Results.

**Table 1:** Scores obtained by Saral for the different items using a severity scale from 0 to 5.

Time course of Saral's autistic symptoms						
Items		Periods				
Situation/skills	Specific focus	I	II	III	IV	V
Upright movement	Speed and quality	3	3	3	1	0
Standing (static)	Symmetry without support				2	2
	Symmetry with hand support				3	3
Sitting on the floor	Dynamics		3	3	1	1
	Symmetry		3	3	1	1
Sitting on the chair	Feet flat	5	4	3	2	1
	Upper limbs	3	2	2	1	0
	Legs	4	4	3	2	1
	Pelvis	3	3	3	2	0
	Verticality of the trunk	4	3	3	2	1
	Vertical movements of the lower limbs	4	3	3	1	
	Duration	4	3	2	0	0
Body games	Proximity/distance		4	4	1	0
Winding	Achievement		5	5	1	0
Squatting	Posture		3	2	0	0
	Standing up				4	1
Bodyball						
Tilt forward	Feet (position, support)	3	3	1	1	0
	Parallel hands	3	3	2	1	0
	Hands (symmetrical push)	5	3	2	1	0
	Head in line	4	3		1	0
Sitting	Balancing trunk	3	3		1	0
	Hands in support	3	2		0	0
	Bounce/verticality		2		0	0
Standing	Hitting the ball	5			0	
	Appropriate use of hands	3	2	2	0	0
	Exchanges	3	3	2	1	0
Trampoline						
Autonomous jumping	Achievement	1	1	1	1	1
	Hand support	3	3		2	2
	Jump propulsion (verticality)	3	3		3	2
	Feet/symmetry	2	2	2	1	1
With human assistance	Quality of upper limb support for propulsion	4			3	1
	Up/down coordination	3		3	2	2
Use of hands						
	Relational grip	5	4		2	1
	Fine motor skills	3	2	2	1	1
	Graphics	3	3	2	1	
	Clapping hands			3	2	1
	Use of thumb		5	2	2	1
Communication						
	Expressive language	4		3	2	2
	Receptive language	4		3	2	1
	Nonverbal communication	2	2	1	0	1
	Use of pictures	3	2	1	0	0
Behavior						
	Impulsivity	4	2	2	2	1
	Joint attention	3	2	2	0	0
	Interactions	3	2	1	1	0
	Eye contact	3	2	2	1	0
	Stereotypies	4	3	3	2	1
	Imitation		3	2	1	
Counting					4	1

Each period lasted 4 months. Upright movement; the tendency to run and walking characteristics are assessed. Sitting on the floor; dynamic adaptations and the search for balance in the sitting position are assessed. Body games; we evaluate the child's ability to accept physical closeness. Winding; quality of forward bending of the trunk during different activities (putting on the shoe, boat games, somersaults).

**Table 2:** Scores obtained by Bahiya for the different items using a severity scale from 0 to 5.

Time course of bahiya's autistic symptoms							
Items		Periods					
Situation/skills	Specific focus	I	II	III	IV	V	VI
Upright movement	Equine walking	5	5	4	3	1	1
Standing (static)	Symmetry without support	5		4	4	3	1
	Upper/lower body rotation	5		4	4	3	1
	Symmetry with hand support	5		4	4	3	1
Sitting on the chair	Feet flat	5	5	4	4	2	1
	Upper limbs	4	3	3	3	1	0
	Legs	5	4	3	3	1	1
	Pelvis	4	4	4	3	1	0
	Verticality of the trunk	3	3	3	3	1	1
	Trunk rotation	4	4	2	2	1	1
	Vertical movements of the lower limbs	4	4	3	2	2	1
Body games	Boat game		4	4			2
Windings	Achievement			4	4		
Squatting	Position	5		3	3	2	2
	Standing up			4	3	2	2
Bodyball							
Tilt forward	Feet position, support)	4	4	3			0
	Parallel hands	5	5	3			0
	Hands (symmetrical push)	5	5	3			1
	Head in line	5	5	3			0
Standing	Tap the ball	5	4	4			2
	Appropriate use of hands	4	4				2
	Exchanges	4	4				2
Trampoline							
Autonomous jumps	Achievement	5	5	3			1
	Hand support		3	3			1
	Jump propulsion (verticality)	5	5	3			1
	feet/symmetry	5	5	4			1
With human assistance	Quality of upper limb support for propulsion	4	3	2			1
	Up/down coordination		4	3			2
Use of hands							
	Relational grip	4	3	2	1	1	1
	Fine motor skills	4	3	3	2	2	1
	Graphics	4	3	3	3	2	
	Clapping hands			3	2	2	1
	Thumb use	5	5	4	3	1	1
Around the bath							
Undressing/dressing	Upper body			3	3	2	1
	Lower body	4	3	2	2	2	0
	One-legged balance				3	2	1
	Autonomy	4	4	4	3	2	1
In the bath							
Upper limb involvement	Use of fingers				4	2	2
	Use of thumbs				5	1	1
	Upper limb as support				4	2	1
	Jet exploration				4	1	0
	Two-hand coordination				3	2	1
Handling/weight	Holding object in hand				5	1	1
	Changing hands				5	2	0
	Forearm support				5	1	0
	Variety of play				5	3	2
Lower limb involvement	Sole support on the bottom				5	4	2
	Sole support on the wall				5	3	1
	Back of foot support				5	3	1
	Supination				5	4	0
	Tonic variation				5	4	2
Involvement of the oral sphere		5	5		5	3	1
Twisting of the body					4	3	1
Incurvation of the trunk						5	1
Lying down					4	2	1
Communication							
	Expressive language	5	4	4	4	3	3
	Receptive language	4	3	3	3	2	2
	Nonverbal communication	5	4	3	2	2	2
	Use of pictures	4	3	3	2	2	
Behavior							
	Impulsiveness	5	3	3	2	2	1
	Self-aggression	5	3	0	0	0	0
	Joint attention	4	4	3		2	1
	Interactions	4	3	3	2	2	1
	Imitation	4		3	2		2
	Eye contact	4	4	3	3	2	2
	Stereotypies	4	4	4	3	1	0
	School activities	5			4	3	

Each period lasted 4 months.

## Saral

### Posture and locomotion

At first glance, Saral appeared to have good gross motor skills. However, he was restless, moved at a rapid pace, and exhibited postural peculiarities.

**Figure 1:**
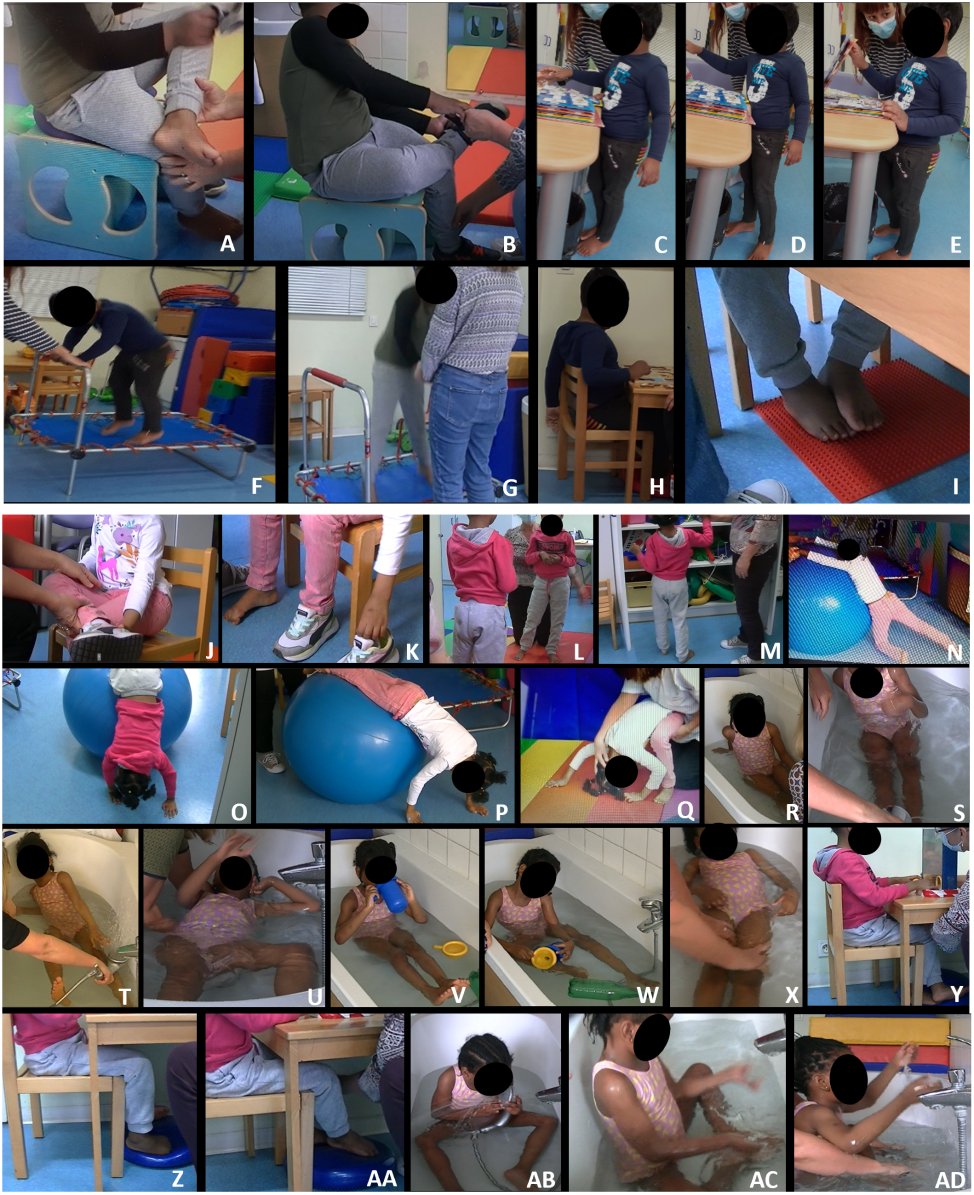
Photographs illustrating Saral's **(A–I)** and Bahiya's **(J–AD)** postural and motor peculiarities. **(A,B)**, Sitting position; placing the left foot on the opposite knee causes the foot to lose support on the ground **(A)** and the trunk to tilt **(B)**. **(C–E)**, Asymmetric sole support in standing position; support is mainly on the left lower limb, the right foot is partially placed on the left **(C)**; note the torsion of the shoulders to the left **(C,D)**; the support of the left hand improves the orientation of the trunk **(E)**. **(F,G)**, on the trampoline; there is no coordination between the upper and lower part of the body; the torso is not straightened by seeking verticality but by transferring the weight of the body to the hands. **(H,I)** Activities at the table; the sitting position is asymmetrical: the pelvis tilts forward and one arm passes behind the back of the chair **(H)**; bilateral sole support begins, but one foot rests on the other and the knees remain abducted **(I)**. **(J)** In the sitting position, placing the right foot on the left knee causes the pelvis to tilt to the right and the trunk to tilt to the left. **(K)** Notice the flexion of the toes and the supination of the foot. **(L, M)** Standing; the sole support is asymmetrical and the pelvis is twisted. **(N–P)**, on the large ball; note the lack of sole support and the torsion of the trunk **(N)** and the asymmetric support of the hands on the ground **(O,P)**. **(Q)** Rolling forward; notice the asymmetric position of the upper limbs. **(R,S)** In the water; one hand is used for support. **(T)** Support on one arm, torsion of the trunk and feet in supination. **(U)**, in the water, Bahiya becomes disorganized as she settles into the decubitus position. **(V,W)** Sitting in the water, Bahiya does not use her lower limbs and loses support on the soles of her feet **(V)** and the verticality of her trunk **(W)**. **(X)**, in decubitus position, lateral flexion and progressive torsion of the trunk when the adult pushes on the longitudinal axis. **(Y,Z) (AA)**, sitting at a table; note the succession of sole supports which remain asymmetrical with supination of the foot **(Z)**. **(AB)** Handling an object in the water; note the support of the upper limbs on the thighs. **(AC,AD)** Playing with the water; neglect of the hands; the fingers are bent, the action is ineffective.

Saral could hardly sit on his pins with his torso upright and both feet on the floor. Asymetry and lack of verticality were worsened when a foot was placed on the opposite knee (Figures 1A, B). He could not tap his feet alternately on the floor in a vertical movement. To put on his sock, he grasped it on the inside of the ankles, but without using his thumb. The outside of the ankle, out of sight, was ignored. Repetition and adaptation of the position using a stool and a dynamic seat cushion encouraged him to straighten his trunk and sole support.

**Figure 2:**
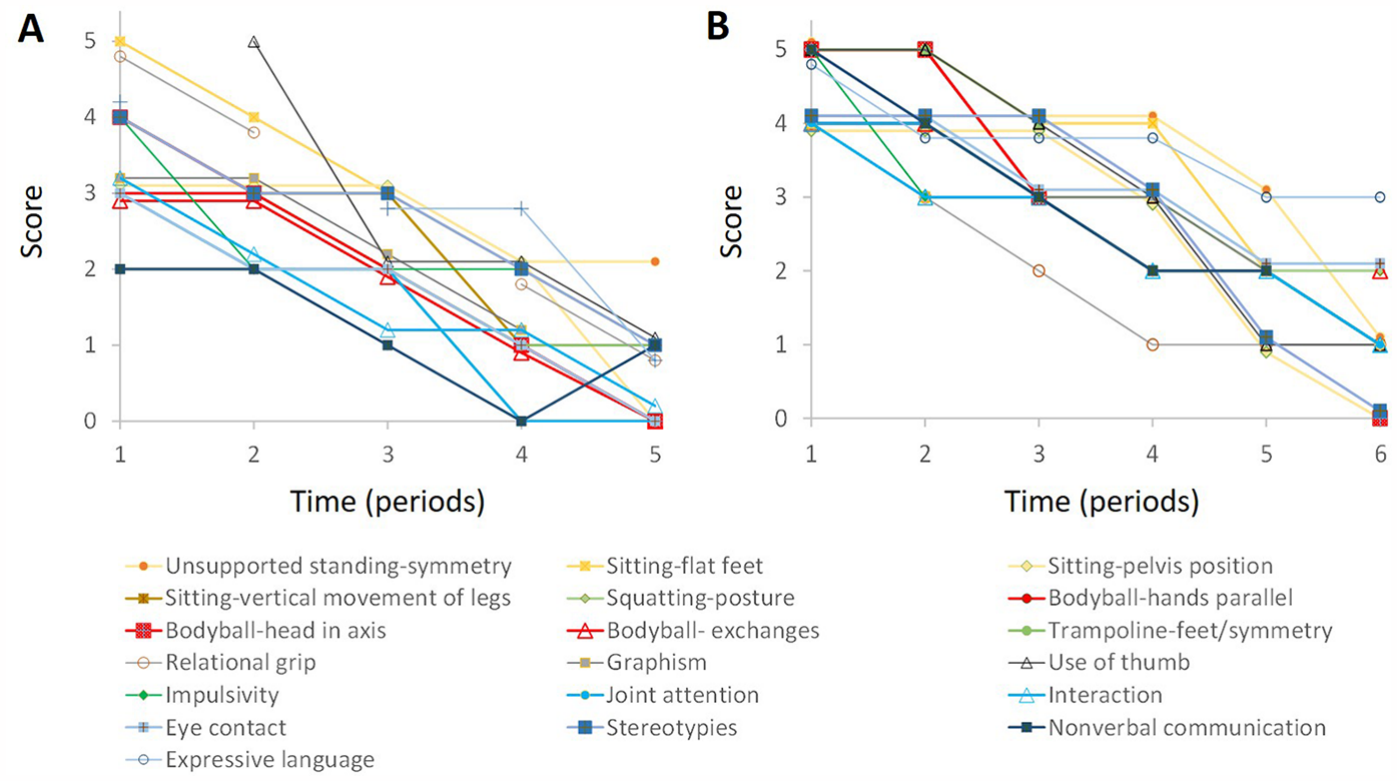
Evolution of the scores as a function of time. For the sake of clarity, only some of the grid items described in Tables 1, 2 are shown and somes curves were slightly shifted upward or downward by 0.1 or 0.2 unit. **(A)**, Saral. **(B)**, Bahiya. Note the synchronous improvement in the different items.

Even in the static standing position the posture was asymmetrical. One foot overlapped the other or was shifted backward; the body was sometimes supported on an upper limb (Figures 1C–E). On the trampoline, Saral bounced his lower body without coordination with his upper body (Figure 1F). In addition, his upper limbs were alternately uninvolved or tense (Figure 1G).

The lack of spatial cues was evident when Saral was placed in the procubitus position on the large ball and tipped forward. Instead of pushing off the floor with both hands, he supported himself on his head with his hands trapped under the ball. When he could free his hands to support himself, they were wide apart with the fingers pointing toward the ball. He pushed the large ball with the backs of his hands; his feet were not parallel, causing an imbalance in his pelvis. Later, when he could organize symmetrical foot support, he was able to push or throw the ball with his palms. Until period IV, windings and movements in the sagittal plane were difficult to achieve as was the vertical uprighting from the squatting position.

### Hand use and fine motor coordination

Initially, Saral never gave his hand to the adult (hereafter referred to as relational grip). His fingers, especially the thumb, did not close over the adult hand. During fine motor activities, it was observed that sitting promoted distal involvement and visuomotor coordination. In contrast, the static standing position made it difficult for Saral to perform complex activities, especially when leaning on the desk with his upper limbs (Figures 1C–E).

Initially, Saral's use of equipment was repetitive and accompanied by upper limb and facial stereotypies. His posture was twisted, with his feet resting on the outer edges of the sole or one foot overlapping the other (Figures 1H,I). He needed to be helped to regain a balanced posture so that he could play more complex games and maintain his attention. Up to period V, Saral did not use his thumb to manipulate small objects.

### Communication, language and behavior

In period I, Saral said a few words spontaneously and in echolalia. He expressed his requests by pointing, pulling the adult's arm, or using pictures. Communication skills developed gradually. In period V, Saral expressed his requests verbally using single words, sometimes in echolalia, pictures, or gestures. Eye contact was present during exchanges. Impulsivity and hyperactivity decreased during period I. Gradually, stereotypies, joint attention, and relationships improved, as shown in Table 1 and Figure 2.

### Correlation between data

Figure 2A shows that, for all items, there was a progressive decrease in scores. This change was consistent with the reduction in the score obtained with the CARS (36 at the beginning of the study and 26 at the end). The different curves are approximately parallel indicating a strong correlation between the scores obtained for the different items. In other words, the progressive disappearance of postural abnormalities was accompanied by an overall improvement in autistic symptomatology.

## Bahiya

To describe Bahiya's symptomatology, we scored 67 items, as shown in Table 2 and Figure 2B.

### Posture and locomotion

Initially, Bahiya had fairly good motor skills, allowing her to walk, although often on tiptoe, climb stairs, and pedal with assistance. However, we soon noticed peculiarities.

When Bahiya was sitting in a chair, the support was only on one ischium and the hands, and also on the back when the pelvis slipped forward. When the posture was straightened, it was rotated around the vertical axis, with the feet in supination, the thighs turned to one side and the torso and gaze turned to the other side. Placing one ankle on the opposite knee further tilted the posture (Figure 1J).

During the first 12 months, Bahiya could not tap her feet alternately on the floor, nor she could clap her hands vertically or horizontally on the therapist's hands. From period I to V, Bahiya was constantly twisted around the vertical axis, with or without upper body support, when she was in the standing position (Figures 1L,M). In period I, in procubitus on the bodyball, she could be pulled forward, but contorted to return to her feet (Figure 1N). During the forward tilt, she twisted around the vertical axis and her hands barely touched the ground. She did not seem to have any representation of the longitudinal and anterior-posterior axes. When she could touch the ground, the support remained asymmetrical with a twisted torso (Figures 1O,P).

The notion of verticality seemed to develop in period III; she began to jump on the trampoline, but the feet were not parallel, their support was not synchronous, and the coordination between the upper and lower body was fragile.

Initially, Bahiya could only get up from the squatting position by leaning on her hands. The standing position was built on an asymmetrical support of the feet with a twist starting from the pelvis. The somersault was hardly achieved and the symmetrical support of the hands was disorganized when the head touched the mat (Figure 1Q).

At the end of Period III, postural abnormalities were still very present and Bahiya's behavior was still rigid and repetitive. To encourage her to modify her motor organization, we started working in the water. In the beginning, her sitting position in the tub was not balanced. She tilted in all directions and had to use her hands to regain her position (Figures 1R,S). Any change in position or emotion caused her body to twist (Figure 1T). When Bahiya was placed in the supine position, she was stiff, with her pelvis twisted (Figure 1U). With training, she reinforced the sole support and stabilized sitting, but the lower limbs rose to the surface as she handled the bottle (Figures 1V,W). As she progressed in the balance along horizontal and sagittal planes, we oberved better involvement of upper limbs and thumbs for grasping. Still, Bahiya's body gradually twisted during passive rocking movements (Figure 1X). With more training, she could use the sole support on the bottom of the tub to stabilize her position and use her hands to manipulate rather than to restore balance.

### Hand use and fine motor coordination

Initially, relational grip was almost absent. It improved gradually but the use of the thumb appeared only in period IV. In Period I her interests were mainly sensory; she had limited imitation skills. In Period II, her sitting posture was still twisted and her feet rested on her curled toes (Figures 1Y–AA). By adjusting the footrests with a flexible tactile pad, she was able to reposition herself on the back of the chair. Apparently, these corrections helped Bahiya to match colors and shapes. Indeed, the speed and accuracy of her responses correlated with the symmetrical balance of her posture. In the bath at period IV, hand use defect was still obvious. To support the shower head, she had to rest it and her forearm on her thigh (Figure 1AB). When she tried to throw water, her upper limbs moved back and forth, but her hands offered no resistance to the water (Figures 1AC–AD). Gradually, as the support of the feet on the tub improved, she could better use her hands and her thumbs for example to hold a bottle.

### Communication and language

During period I, Bahiya did not use pointing, did not make requests, produced syllables and occasionally a few words, and did not respond to her name. Gradually, nonverbal communication improved but expressive language remained limited.

### Sociability and behavior

Initially, eye contact was infrequent and consisted of a sidelong glance accompanied by a rictus. She did not play with other children and participated very little in school activities. To express displeasure, she would tap her forehead on hard surfaces. Self-aggressive episodes decreased significantly during the first months. Gradually, her ability to interact with others and regulate her emotions improved, as did eye contact and stereotypies. Activities (balloon exchange, dinette) remained significantly repetitive and stereotyped until period IV. However, she showed enthusiasm when her body axes were challenged (squatting, trampoline, bodyball).

### Correlation between data

Figure 2B shows the progressive decrease of Bahiya's scores in the different domains and the strong correlation between scores obtained in the different areas. The decrease in the CARS score (39.5 at the beginning of the study and 30 at the end) was consistent with the decrease in the scores obtained with our observation scale.

## Discussion

### Motor impairments in ASD: comorbidity or dimension of autism?

Motor impairments are highly prevalent in ASD and seem to be similar to those found in developmental coordination disorder (DCD) (7–13, 30). However, the exact nature of the motor difficulties (31) and the underlying mechanisms may not be identical (32–35). For example, it was found (31) that, compared to controls, movements were slower and more fluid in ASD and less fluid in DCD.

Some motor impairments seem to us to be more specific to ASD: (i) atypical support; children with ASD often use their hands as pillars when sitting or seek support with the upper body when standing; (ii) lack of dissociation of the girdles, which affects balance; (iii) twisting postures of the trunk, neck, girdles and limbs; (iv) distal predominance of deficits; hands and fingers seem to be partially excluded from action planning; (v) avoidance of the thumb in manipulations or relational grip; (vi) severe deficit in representing the body in space and especially in verticality. Some of these characteristics were previsouly observed in ASD (15, 17, 18). Further research would be useful to determine whether these motor signs are indeed specific to ASD and could be used as diagnostic criteria.

In our clinical experience, motor disorders are always present in children with ASD but sometimes subtle or difficult to detect at first because children avoid situations in which they are in difficulty because of their motor problems.

Overall, the data suggest that children with ASD lack physical reference points, as if they have not yet established a functional “body axis” (36).

### A “body axis oriented” therapeutic approach

So far, therapeutic approaches for autism have not focused on improving gross motor skills. Our approach is based on the hypothesis that the development of the body axis can help children with ASD acquire the spatial and physical reference points necessary for the development of their means of acting on their environment and thus of their cognitive and relational abilities. To achieve this goal, children are placed in situations that encourage them to seek out new supports and explore new postures in order to develop new sensory cues and capacities for action.

In the face of a fine motor deficit, it may be tempting to focus on manipulations. We think it is more effective to help the child develop a better awareness of his body and the space around him. Once he has acquired more functional postures, hand use and fine motor skills can progress rapidly. The therapeutic approach suggested here can be adapted by any therapist and is complementary to other approaches that can help children with ASD acquire means of action and expression. We offer some general recommendations: (1) offer children a variety of activities that involve the different dimensions of the body axis; (2) every detail can be important. We learn the importance of a postural element when its correction improves the child's participation or efficiency; (3) help the child correct his posture by seeking symmetry in the three axes, which allows him to discover verticality and coordination; (4) use the child's sensory peculiarities through various explorations; (5) suggest work in the water when the therapeutic alliance is solid, when the child has already made progress in developing the body axis, and when progress is stagnant; (6) provide a recognizable framework for the sessions, including beginning and ending rituals and the use of appropriate communication.

Our data suggest that postural and locomotor disorders correct themselves synchronously during treatment. In addition, motor skills appear to develop at the same rate as the cognitive and social skills that are affected in ASD (Tables 1, 2, Figure 2). In our experience, the synchronous change of autistic symptoms and motor disorders has been observed in most of the children who have benefited from the therapeutic approach described here. Obviously, this result needs to be validated by studies with a larger number of children.

Our results are consistent with several studies suggesting that motor dysfunction is a central aspect of autism and that therapeutic interventions should be aimed at improving motor performance (9, 10, 30). Theories of embodied cognition suggest that language and conceptual acquisition are grounded in action and perception (37–39), and it has been proposed that a wealth of motor, perceptual, and cognitive features of ASD can be understood in terms of a deficit in perceptual-action integration (37). Eigsti (38) suggested providing motor experiences that could improve embodied processing. Our therapeutic approach may address the weakness of embodied cognition in children with ASD.

### Limitations

An obvious limitation of this study is the small number of children enrolled. Also lacking is a control group of children who would benefit from similar treatment, but without the specific body axis-oriented psychomotricity approach described here. Future work is needed to test our hypotheses with improved study design.

Regarding the observation grid, further work is needed to identify the most relevant items and to test its psychometric properties. The grid should be scored by several investigators to calculate inter-rater reliability.

## Data Availability

The original contributions presented in the study are included in the article/Supplementary Material, further inquiries can be directed to the corresponding author.

## Supplementary material

Supplementary Figure 1 Photographs illustrating the sessions and certain sensory, postural, and motor characteristics. A, sitting in the chair, facing the therapist, with the ankle resting on the opposite knee; note the hand resting on the chair. B, sitting on the chair, the child must tap the floor with the soles of his feet, alternating between the two legs; note the lack of support on the right leg and the movement of the left leg forward and not upward. C, clap on the therapist's hands, both in the frontal plane. D, clap on the therapist's hands, one hand in the frontal plane, the other in the horizontal plane. E, in procubitus position on the big ball; the child does not support himself with his feet on the floor and does not try to stand up. F, G, holding the adult's hand; note the lack of flexion of the fingers and thumb on the adult's hand, which characterizes the lack of relational grip. H, I, sitting on the large ball; lateral oscillations (H) or in the sagittal axis (I). J, jumping on the trampoline; only the right leg is active, the left leg, backwards, is used for support. K, jumping on the trampoline: note the asymmetric and asynchronous support. L, standing up from a squatting position. M, playing with the boat; notice the lack of flexion of the fingers in the relational grip. N, in the bathtub, visual perception of the jet of water as graspable; note the displacement of the thumb. O, in the water; notice the curvature of the trunk when the child is pushed along the longitudinal axis. P, in the water; notice the twisting of the waist and trunk as the child is pushed along the longitudinal axis. Q, in the water; notice the use of the hands to keep the head above the water. R, sitting in the water; notice the use of the right hand and left forearm as supports to use the left hand. S, playing with the water; note the position of the thumbs, which are not in opposition, to hold the bottle.
