# Supplementary material for: Improvement of motor disorders and autistic symptomatology by an approach centered on the body axis: a two-case report
Source: Front Child Adolesc Psychiatry. 2025 Apr 14;4:1451559. doi: 10.3389/frcha.2025.1451559 (PMC12034731; doi:10.3389/frcha.2025.1451559)
Supplement: Supplementary file 1 [file Supplementaryfile1.docx]

**METHODS**

**Patients**

Saral (first names have been changed) was of Indian origin and the youngest of two boys. Parents were unrelated. At home, only the oldest boy was speaking french. Parents had poor French language skills. Saral was exposed to french at school and in our center. M-CHAT was found positive at 18 months by a pediatrician. He was 4 years 7 months old when we met him. At that time, he did not seek interaction with adults and children except in a dysfunctional way by provoking and testing limits. He could not initiate play. It was difficult to maintain interaction with him. He did not share interests and emotions. Verbal and non-verbal communication was impaired. He could name members of his family, colors, numbers from 1 to 10, and a few everyday objects, and also talked in jargon. Echolalia appeared later. He could understand a few simple instructions but did not respond to his name. Eye contact was rare but possible. Pointing had been acquired only recently and was used occasionally but he had no gesture to say yes or no. Joint attention was very limited and imitation was observed only when objects were manipulated. Saral had some gestural stereotypies of the face and upper limbs ; sometimes, he repeatedly manipulated doors, switches and certain toys. His most pervasive behavior was his tendency to run, climb on furniture, or run away. He found it hard to stop an activity, especially if it was visually interesting. His tendency to exert strong pressure on objects or to bite them was suggestive of tactile sensory particularities.

Bahiya was of Nigerian origin and the youngest of three siblings. Parents were unrelated and had poor French language skills and spoke english at home. At that age, Bahiya had limited social interactions. At the beginning of the study, Bahiya was 3 years and 9 months old. She tolerated the presence of children by her side but did not play with them. Previously, she had slapped any child who came near her. Eye contact was rare. She did not respond to her name. Expressive langage was limited to a single word. Non-verbal communication was also very limited : she didn't use finger-pointing or move her head to say yes or no. She had stereotypies of eyes and face and often tiptoed. Repetitive behaviors were observed and she reacted strongly to any change that disrupted her habits. At home, she had a special interest in modeling clay. She also put it in her mouth. Her register of gaming interests was limited. She was hypersensitive to sounds. Strong emotional expressions of anger or distress were often observed

**Psychomotricity session schedule**

The therapist guides the child to develop symmetrical posture, harmonious movements in all three planes and proprioception. Ritualized activities such as taking off the shoes and rhymes at the beginning of the session and putting on the shoes at the end set time limits for the sessions. Some of the activities commonly offered to children are described below.

**-** **Taking off the shoe.** The child sits in the small chair; facing him, the therapist asks him to place his ankle on the opposite knee (Sup Fig. 1A) and then to remove his shoe. As long as the child does not have awareness and correct coordination of his two hemi-bodies, this simple movement modifies the support of the foot on the ground, the balance of the pelvis and the upper body. While removing the shoe, we also observe the organization of the movement and the use of the thumb.

**- The feet are alternately tapped on the floor.** The child sits on the chair. To perform this alternating movement of the legs in the frontal plane, he must shift his weight onto the ischium and perform a movement about a frontal axis passing through the hips, coordinating the two hemi-bodies (Sup Fig. 1B).

**- Nursery rhymes** combine descriptions of posture and limb movement with song and sound. Rhymes are used to develop body representation, imitation, oralization, and interaction skills.

**- Clapping the adult's hands.** The child is seated and asked to clap simultaneously on the therapist's two hands in different positions, symmetrical or not. The gesture requires a representation of the position of the hands along the three body axes, abd combines an impulse followed by a withdrawal movement. It mobilizes the child's interactive abilities (Sup Fig. 1C, D).

**- Putting on the socks at the end of the session.** The seated child is asked to place the heel on the edge of the chair and pull the top of the sock with both hands, passing the arms on either side of the lower limb. The other foot is flat on the floor. To put on the shoe, the child is asked to push the foot vertically without using the hands or twisting the body. The therapist looks for awareness of the lower limbs and the two hemi-bodies, as well as verticality.

**- Procubitus on the bodyball (80 cm in diameter).** The therapist kneels facing the child in the procubitus position on the ball, holding the child's hands and making rocking movements along the longitudinal axis. We observe whether the trunk is in line, the position of the feet when they touch the ground and their support on the ground. Some children avoid placing their feet on the floor (Sup Fig. 1E). We observe how the child holds the adult's hands, which we will call relational grip, and the involvement of the thumbs (Sup Fig. 1F, G). The child's behavior provides information about his or her representation of the body along the longitudinal axis, the coordination between the upper and lower body, and the involvement of the distal extremities.

The therapist then stands behind the child, who remains procubitus on the ball, holding it by the ankles. The therapist rolls the ball forward and encourages the child to place his hands on the floor, lean on the floor and then push it away to return to the starting position with the help of the adult who pulls him backward. The situation brings into play the symmetrical representation of the body and verticality. The position of the head is also observed.

**- Sitting on the bodyball**; the child, held by the lower limbs, is rocked back and forth and then sideways. The position of the trunk, the symmetry of the sitting position and the use of the hands are observed (Sup Fig. 1H, I). With a nursery rhyme (..."walk, trot, gallop") we stimulate increasing jumps. We look for the child's vertical impulse, which essentially involves the trunk. - **On** **the trampoline**, the child shows us his ability to jump and take off independently, which gives us information about the child's perception of verticality and the use of the body axes. We observe whether he uses the support bar, whether this support is flexible or rigid, and whether the body weight moves towards this support. By giving him his hands, we assess the relational grip and the support that the child uses. We assess the symmetry of the sole supports, the strength of the impulse and the synchronization of the lower limbs (Sup Fig. 1J, K). We suggest rotations to see if they involve the whole body or if there is a torsion between the upper and lower body.

**- Standing upright from a squatting position** stimulates the longitudinal axis and verticality. Standing on the floor behind the child, we bring the child into a squatting position. The child's feet should be parallel with the heels on the floor. Any support with the upper limbs, twisting of the body or digitigrade support must be ruled out. What we are looking for is the feeling of straightening (Sup Fig. 1L).

**- Forward roll.** The therapist sits on the mat on his heels. The child sits on her thighs. We help him to position his two feet parallel, then his two hands in front of his feet, parallel to each other, then we help the child to roll forward. The child's ability to roll up is observed as soon as he puts his hands on the floor.

- For **the boat game**, the child and adult sit facing each other, legs stretched out on either side of the other's body, and take turns pulling each other with outstretched arms to the rhythm of a nursery rhyme. We end with a passive roll and a sit-up. We look for interaction, relational grip, rolling up, feeling the hinge between the upper and lower body, balanced lengthening of the body (without supporting the hands and without twisting), and verticality (Sup Fig. 1M).

As part of therapy, children are often asked **to work in the water**. This comes into play when there are limitations in the progress of the construction of the body axis and in the cognitive and relational achievements. First of all, it is necessary to gain the trust of the child and the parents and to be able to rely on a representation, even a partial one, of the supports and axes of the body. This is done in a bathtub. The child enters the empty bathtub in a bathing suit and is invited to sit down. In this sensory experience we observe how the child perceives the jet of water as it fills. The child often perceives the flowing water as a stick and tries to grasp it using a variety of strategies. In the water, the child's support and balance are modified. The autistic body organization becomes more apparent. We approach the notions of transparency, depth, gravity, unity of the body, balance, grouping, elongation, and the various hinges, spatial representations that favor the representation of the body axes (Sup Fig. 1N-Q) and the involvement of the extremities (Sup Fig. 1R). Some objects, such as a bottle, a pitcher and a funnel, can then be offered to manipulate the water and test its resistance (Sup Fig. 1S). The child remains in the tub as the water is drained. The child is then wrapped in a sheet and carried out of the tub. After listening to a nursery rhyme that names various parts of the body, the child sits on the lap of an adult who applies pressure to the parts of the body mentioned. Work in the water ends at the child's explicit request or when symbolic and interactional play has developed.

**Evaluation**

Each item was given a score on a scale from 0 to 5. Scoring was done by SP. For each four-month period, she assigned an "average" score.

5: Pervasive and irrepressible autistic features. Severely maladaptive responses or behavior on a consistent basis. Maximum functional impact.

4: Autistic features often strongly expressed. Responses or behavior frequently severely maladaptive. Very significant functional impact.

3: Moderately disabling autistic features. Severely inappropriate responses or behavior in several situations. Significant functional impact.

2: Inconsistent autistic features with little disabling effect. Appearance of new skills allowing some adaptations. Moderate functional impact.

1: Discrete autistic features. The child uses his skills to adapt. Low functional impact.

0: No autistic features. The responses and adaptations are consistent with the age group. There is no functional impact.
