# Supplementary material for: Improvement of motor disorders and autistic symptomatology by an approach centered on the body axis: a two-case report
Source: Front Child Adolesc Psychiatry. 2025 Apr 14;4:1451559. doi: 10.3389/frcha.2025.1451559 (PMC12034731; doi:10.3389/frcha.2025.1451559)
Supplement: Supplementary Figure 1 — Photographs illustrating the sessions and certain sensory, postural, and motor characteristics. A, sitting in the chair, facing the therapist, with the ankle resting on the opposite knee; note the hand resting on the chair. B, sitting on the chair, the child must tap the floor with the soles of his feet, alternating between the two legs; note the lack of support on the right leg and the movement of the left leg forward and not upward. C, clap on the therapist's hands, both in the frontal plane. D, clap on the therapist's hands, one hand in the frontal plane, the other in the horizontal plane. E, in procubitus position on the big ball; the child does not support himself with his feet on the floor and does not try to stand up. F, G, holding the adult's hand; note the lack of flexion of the fingers and thumb on the adult's hand, which characterizes the lack of relational grip. H, I, sitting on the large ball; lateral oscillations (H) or in the sagittal axis (I). J, jumping on the trampoline; only the right leg is active, the left leg, backwards, is used for support. K, jumping on the trampoline: note the asymmetric and asynchronous support. L, standing up from a squatting position. M, playing with the boat; notice the lack of flexion of the fingers in the relational grip. N, in the bathtub, visual perception of the jet of water as graspable; note the displacement of the thumb. O, in the water; notice the curvature of the trunk when the child is pushed along the longitudinal axis. P, in the water; notice the twisting of the waist and trunk as the child is pushed along the longitudinal axis. Q, in the water; notice the use of the hands to keep the head above the water. R, sitting in the water; notice the use of the right hand and left forearm as supports to use the left hand. S, playing with the water; note the position of the thumbs, which are not in opposition, to hold the bottle. [file Supplementaryfile2.pdf]

RESULTS –extended version

SARAL

We assessed around fifty items and their evolution over the course of treatment to describe Saral's symptomatology. For each four-month period, we assigned an "average" score to each item on a scale from 0 to 5, with 5 representing the maximum severity. The values are provided in Table 1.

Posture and locomotion

At first glance, Saral appears to have good gross motor skills. He walked when he was 1 year old. The soles of his feet come to rest when he walks. He walks up and down the stairs, alternating his feet. However, he is restless, with a fast, hurried pace. He takes risks by running away and jumping from one support to another. He never gives the adult his hand. In other words, he has no relational grip. His parents hold him by the wrist. When he is not upright and the longitudinal axis deviates from the vertical, it is clear that he has postural peculiarities. *Sitting on the chair.* At the beginning (periods I and II), the lack of organization of motor skills around the body's axes is clearly evident in the sitting posture by asymmetry, twisting around the longitudinal axis, and a lack of verticality (score (S)=4). On a small chair, his pelvis slides forward, one arm passes behind the backrest, the knees spread apart, the feet touch or overlap (S=5). Eye contact is inconsistent (S=3). He is unable to tap his feet alternately on the floor in a vertical movement, but tilts his torso backward and throws his legs forward (S=4). When the right foot is placed on the opposite knee, the left foot loses support and crosses the longitudinal axis (Fig. 2A). His torso tilts to the left as he carries his weight on his right buttock (Fig. 2B) (S=3). Repetition, imitation of the therapist and physical adaptation of the position using a stool and a dynamic seat cushion encouraged him to straighten his trunk (S=3) and

sole support (S=3), which could be observed from period III onwards. The movement of tapping the ground with each foot becomes more vertical (S=3). In period V, the hip no longer collapses (S=0) when the foot lands on the knee of the opposite limb. In certain actions, the flat position of the feet becomes automatic. While his knees were still abducted, he gradually became aware of his lower limbs (S=2 then S=1), first with a vibrating cushion that he had to press between his knees with his hands, then without any equipment. Through imitation, he is able to reproduce push/release coordination (adduction/abduction). During fine motor activities, it was observed that sitting promoted distal involvement and visuomotor coordination. Saral began to generalize a balanced posture with his feet flat and parallel on a sensory tile. Sole support became more effective due to the distance between the two feet (S=1).

In period I, to put on his shoes at the end of the session, Saral, sitting on a small chair with his knees abducted, pulled his foot in front of him with his hands. His torso tilted backwards and the foot on the floor lost its support in a rather unusual adduction movement (S=4). In period II, even with the help of the psychomotor therapist, Saral finds it difficult to sit on his pins, with his torso upright (S=3) and both feet on the floor. He grasps the sock at the end of his foot on the inside of the ankle with his clawed fingers, but without using his thumb (S=5). The outside of the ankle, out of sight, is ignored. The other foot is lifted off the ground, adducted. Saral balances on one buttock (S=3). In period III, sitting on a stool to prevent the pelvis from slipping, Saral leans forward to pull on his sock, but he cannot support his trunk, which is leaning on his thighs (S=3); the other foot loses its support on the floor and folds under the chair. He is still unable to respond to the instruction to push his foot vertically into the shoe. He does not use his thumb to close the grip. In period IV, Saral had a better sense of the volume of his foot and, with his heel on the edge of the stool, pulled on the sock on the top and inside

of his foot. In period V, Saral showed better body awareness and a good ability to roll up (S=0). The trunk remains close to the longitudinal axis (S=1) and he can pull the sock with one hand on either side of the knee. The foot stays on the ground. When he leans forward to adjust the heel of his shoe, his trunk no longer collapses on his thighs and the mobility of his upper limbs is preserved (S=0).

Standing position. At the beginning of the treatment, Saral's restlessness severely limits the use of the static standing posture. When it becomes possible to observe this posture (periods IV and V), it remains asymmetrical (S=2) and Saral's ability to pay attention and perform complex tasks is limited. One foot overlaps the other or is shifted backward; the body is supported on one foot (Fig. 2C-E) and sometimes on an upper limb (S=3).

Trampoline. In Period I, Saral is very attracted to the trampoline and uses large arm movements to propel his jumps (S=1). His shoulders are tense and his lower limbs are spread wide to maintain balance. He avoids facing the therapist. When he holds the bar, his upper body stiffens; Saral looks down at his feet with his arms outstretched. He bounces his lower body without coordination with his upper body (Fig. 2F) (S=3). When the adult's hands are offered to help him, he cannot grasp them (no relational grip) (S=5). In addition, his upper limbs are alternately uninvolved (forgotten, relaxed) or tense, like the rest of his body (Fig. 2G) (S=4). Although an evolution was noticed until the end of the treatment, Saral maintained the same motor pattern characterized by a lack of synchronization between the upper and lower body involving the vertical dimension of the body axis (S=3 and then S=2). As in the static standing position, the support of the hands contributes to the lack of verticality.

Bodyball. In period I, the lack of integration of the body axis was evident when Saral was placed in the procubitus position on the large ball and tipped forward. Instead of pushing off the floor with both hands, he supports himself on his head with his hands trapped under the ball (S=5).

He then releases one hand to support himself and twists his body (S=3). When the two hands are supported on the ground, they are very far apart and the wrists are turned outwards, pointing the fingers towards the ball. This rigid, columnar support does not allow him the vertical thrust necessary to stand up (S=3). In period III, the hands are immediately positioned as pillars and then come together in their movement towards the ball in order to straighten up vertically (S=2). He can still sometimes show his refusal to act by positioning himself in a twisted position (leaning on one shoulder, one cheek and the opposite hand). In period IV, the support is on two parallel hands (S=1), although he may sometimes repeat the path that leads to this symmetrical support, which allows him to push his body along the longitudinal axis. In period V, the uprighting is active, with support from one half of the body to the other; the hands are close to the other and the wrists are no longer in rotation (S=0).

On the bodyball, in period I, Saral did not use his hands to hold onto the hands of the psychomotricist when he was pulled by his hands along the longitudinal axis. He began to participate in the relational grip in Period II (S=4).

Sitting on the ball, in periods II and III, Saral is still often twisted (S=3); his gaze is not focused. Slowing down the rhythm of his lateral oscillations helped him to become aware of the discomfort and to correct the verticality of his trunk to remedy it. During period V, the coordination between the two hemi-bodies becomes automatic, allowing the torso to stand upright (S=0). In periods I and II, during a short exchange game, Saral pushes the bodyball away with the back of his hand (S=3, then 2). Unmotivated, he wiggles; his feet are not parallel, causing an imbalance in his pelvis. In periods II and III, he begins to enjoy exchanging the bodyball for a rebound. He uses both upper limbs to push or carry the ball and to throw it, which forces him to place his feet correctly and to use the verticality of his body axis (S=2). In

period IV, the exchange with three players becomes possible (S=1). He addresses the ball to the person named by the adult and repeats the first name.

General dynamic coordination, squatting and rolling. In period II, the therapist suggests a "fight" game. He has to resist and defend himself on the mat when he is caught and tickled. He loses his speed and agility and says "no, no, no", showing his need for distance (S=4). The use of the gymnastic ribbons puts the other person's body at a distance. The game creates an alternation of excitement and immobility, followed by the appearance of guided movements and imitation, as well as a regression of excitement in period III (S=3). In period IV, he laughingly accepts the "fights" (S=1) as well as the windings (S=1), such as the squatting position (S=0) to pick up an object, the roll and the boat game which take place on the sagittal axis. The relational grip is also established and becomes automatic in period V, as is the vertical uprighting from the squatting position, which no longer requires the participation of the upper limbs (S=1). With joint attention in place, he becomes a player in the boat game.

Fine motor coordination and adaptation to objects

Initially, Saral is attracted to everything he sees and manipulates objects impulsively and forcefully. He tramples toys to make noise. His use of equipment quickly becomes repetitive. Upper limb and facial stereotypies then appear.

In period I, sitting in front of the small table, Saral quickly performs embedding activities. His posture becomes disorganized, with his feet resting on the outer edges of the sole or one foot overlapping the other, his knees abducting and one arm escaping behind the backrest (S=3). He needs to be helped to regain a balanced posture so that he can play more complex games (Fig. 2H-I). With a seat cushion under his buttocks and a vibrating cushion under his feet, Saral's trunk straightens, freeing his upper limbs. He is given the opportunity to use a small

fishing rod to catch embedded parts. He does not use a supporting hand and an active hand, but a rigid grip on the rod with both hands or with one hand and the head (S=3). In period II, the feet lie flatter but the knees remain largely abducted (Fig. 2J). He uses the fishing rod with one hand. He completes the first two boards of a series of bead algorithms and uses two-hand coordination to thread them onto the stick (S=2). In period IV, Saral is able to adjust his sitting posture independently when he is motivated by the proposed activity. As a result, his gestural organization is fluid when faced with a puzzle of complex geometric shapes. Two-hand coordination is effective and may cross the sagittal axis. His gaze participates synchronously without dropping out. In this period, as in the previous one, the activities become more varied and complex (sand, magic trails, volume puzzles...) (S=1). It should be noted, however, that Saral does not use his thumb to move the magnet that guides a figure on the board (S=2). As explained above in the postural and motor domain, the static standing position in front of the desk makes it difficult for him to perform complex activities. His unbalanced posture prevents him from maintaining his attention.

His graphic skills also developed favorably. In period I, Saral scribbled poorly, reproducing dots and curled shapes. He tried unsuccessfully to draw letters (S=3). In period II, he scribbled strongly and reproduced some vertical lines. He covered the eyes of a drawn man (S=3). In period III, he was interested in writing his first name and let his hand guide him (S=2). In period IV, Saral's drawings were enriched with circles (S=1).

Communication and language

In period I, Saral says a few words spontaneously and repeats them in echolalia (S=4). Saral expresses his requests with proto-imperative pointing or by pulling the adult toward what he wants. He is able to use pictures to express requests by picking them up and pointing at them

(S=3). He can then address them to the adult. In period III, communication with pictures develops and he uses them independently (S=2). He uses gestures to ask for help (S=1). His eyes participate in all his requests. But eye contact is inconsistent (S=2) during activities at the table and especially face to face. His spoken language has been enriched by several words (S=3). When looking for his mother, he is able to say "Where is Mommy? ». In period IV he points to different objects and says what color they are. When offered an activity other than the one he requested, Saral responds "ok, ok, ok!" and accepts the adult's choice. Towards the end of this period he responds to verbal instructions only (S=2). In front of the mirror, he begins to mimic anger (S=1). In period V, Saral expresses his requests verbally using word phrases (S=2), pictures (S=0) or gestures (S=1) and uses echolalia to support his requests. This expression remains factual or material. He responds to simple verbal instructions, gestural instructions, and pictures. Saral associates certain sounds with the pictures he points to or gives at our request. He names them only in echolalia. His gaze is directed and participates in exchanges (S=0). His facial expression has improved.

Behavior

While he was impulsive and hyperactive, Saral's behavior calmed down after about ten sessions (S=4) in Period I. By adapting to his pace and using his interests, such as motor games, his provocations decreased and he accepted the work environment. In period II he moves more slowly, taking his time for each activity (S=4). At home, it is still difficult to maintain the educational framework. When he moves towards the room, he gives a limp hand. His fingers do not close over the adult's hand (S=4). Beginnings of joint attention appear, especially in motor games and bead algorithms (S=2). Eye contact is more frequent in interactions (S=2). Stereotypies are less generalized (S=3). At school he participates with the group in many of

the activities offered. In period III, Saral's behavior at school deteriorated with his change of class. He shows aggressive gestures. In the unit he mimics expressions of anger that he has provoked in his family (S=1). Joint attention appears in some activities (S=2), but the gaze is less directed and the behavior remains impulsive (S=2). He gives his hand to his mother but cannot yet close his fingers to hold it. In period IV, Saral's progress becomes more visible and begins to be more widespread. Joint attention is good (S=0). Saral seeks relationship and attention. He makes requests. He still has difficulty accepting frustration at home. In Period V, Saral continues to make progress in all areas. The school decided to move Saral to the primary school. He walks calmly to the sessions and holds hands with the adult, who still has to remind him sometimes to squeeze his fingers to hold hands (S=1). Impulsive movements have disappeared in places where he is aware of the environment (S=1). His concept of time remains immature. The concept of "after" is not generalized, which leads to frustration. His relationships with others are still characterized by a search for reactions, but they are improving in frequency and quality. Physical contact is now welcomed. He may seek to be cuddled or show tenderness at other times. Stereotypies have become less frequent and less pervasive (S=1).

Correlation between data

Figure 4A shows the changes in scores obtained for Saral in different domains (posture and motor skills, social interaction and communication, behavior). For all items, there is a progressive decrease in scores. This reduction in the severity of the scores obtained with our observation scale is consistent with the reduction in the score obtained with the CARS (36 at the start of the study and 26 at the end). The fact that the different curves are approximately parallel indicates a strong correlation between the scores obtained for the different items. In

other words, the progressive disappearance of postural abnormalities is accompanied by an overall improvement in autistic symptomatology.

BAHIYA

To describe Bahiya's symptoms, we scored 67 items. The scores obtained for each 4-month period are shown in Table 2.

Posture and locomotion

Bahiya has fairly good motor skills, allowing her to walk, although often on tiptoe, to climb stairs by holding onto the handrail, and to pedal with assistance. However, we soon noticed some peculiarities in the situations described below.

Sitting in a chair. In the beginning (Periods I and II), Bahiya sits on a small chair from the side, with one hand on the seat. The support is only on one ischium and the hands, and also on the back when the pelvis slides forward. When the posture is straightened, it is rotated (S=4), with the feet in supination (S=5). The torso and gaze are turned to the right. When one ankle is placed on the opposite knee, the knee tilts into adduction, the foot on the ground turns inward; the curled toes protect the sole of the foot from contact with the ground, the hip collapses (S=4), and the torso flexes to the opposite side (Fig. 3A) (S=4). She is independent when it comes to taking off her shoes, but we have to intervene to help her maintain a balanced posture in relation to the body axes. In period III, the knees are no longer adducted, but the feet remain supinated (Fig. 3B). The posture varies according to the quality of his presence. The arms still sometimes pass behind the backrest (S=3).

During the first 12 months, Bahiya can only passively tap his feet alternately on the floor. As soon as the foot touches the floor, the movement is slowed down (S=4). Contact is made first with the toe and then with the heel. By arranging her seat with a stool, a cushion and a wooden tray to rest her feet on, she manages to tap one foot on the floor a little stiffly at first, then both feet alternately and then together (S=3 at 9 months). There were fewer twisting

movements of the trunk (S=2). Her participation in nursery rhymes also improved. She begins to clap her hands vertically or horizontally with the therapist's two hands, whereas before she could only grasp them delicately (S=3). In period IV, clapping with the adult's hands remains a simple contact without impulse; however, she can organize herself in space to adapt to their symmetrical and asymmetrical positions, except when the palms are facing down. For Bahiya, as for many autistic children, upward movement is difficult because they have not developed the notion of verticality. The progressive improvement of the postural characteristics continued until period VI.

Standing position. In Period I, in the static standing position, Bahiya is constantly turning, with or without upper body support (Fig. 3C, D) (S=5). It evolves slightly in periods III and IV (S=4), then markedly in periods V and VI with work in the water (S=3 then 1). The digitigrade gait followed the same pattern. In the first three periods, standing in front of the bodyball, Bahiya has great difficulty hitting it (S=5 then 4). During the exchanges she is able to catch the ball but not to throw it back (S=4). An outline of this movement appears (S=2) when she is able to position her feet flat and parallel (S=1) in period VI.

Bodyball. In period I, in procubitus on the bodyball, she allows herself to be pulled forward, but contorts to return to her feet (Fig. 3E) and participates little in the relational grip. During the forward tilt, Bahiya twists. She does not participate in any uprighting, and her hands have difficulty making contact with the ground. With one hand raised backwards, she twists to get out of this position (S=5). At this point, she does not seem to have a representation of the longitudinal and anterior-posterior axes. In period II, stimulated by slight lateral oscillations of the ball, Bahiya places her hands on the floor, but without taking support (Fig. 3F) (S=5). In period III, her hands are first placed asymmetrically on the ground (Fig. 3G), twisting the body,

then sometimes more parallel (S=3), allowing her to push in line with the spine (S=3). An almost complete correction is observed in period VI.

Trampoline. Until period II, Bahiya lies down on the trampoline. She does not jump and tries very little to bounce (S=5). Her pelvis is turned to the left (S=4). Gradually, thanks to the impulses given by the therapist, she let herself jump by holding on to the bar (S=3). In period III, she still leans heavily on the bar, her body away from the support, and jumps on her toes (S=4). Then she moves closer to the bar and jumps alone (S=3). When we give her our hands, her upper body is tonic and she seeks propulsion (S=2). The idea of verticality seems to be developing (S=3). However, the feet are not parallel and their support is not synchronous (S=4). In period VI, there is a fairly good correction of most elements (S=1), but the coordination between the upper and lower body remains fragile (S=2).

Squat and roll. At first, Bahiya can only get up from the squatting position by leaning on her hands and passing through equinus. The standing position is erected on an asymmetrical support of the feet with a twist starting from the pelvis (S=4). Then she straightens (S=2), reducing the torsion and the support on the hands. The somersault remains difficult (S=4). The symmetrical support of the hands is disorganized when the head touches the mat (Fig. 3H). The support of the feet weakens during the roll and causes a collapse to the right.

Work in the water. Despite the progress observed at the end of Period III, twisting and asymmetrical postures were still very present. In addition, Bahiya's behavior was still rigid and repetitive. To expose Bahiya to new sensory experiences and to encourage her to modify her postural and motor organization, we suggested working in the water.

In the tub, in period IV, the first few times it is necesary to help her sit down. She squats on her toes and holds the sides of the tub. When she sits down, she is curious but worries about the jet of water coming out of the faucet. Her interest in the water turns into a search for

water to drink (S=5). With her body twisted from her pelvis (S=4), leaning on her hands (S=4), she drinks. She is then placed facing the jet of water. Sitting with her back against one wall and her feet against the other, Bahiya is invited to explore the flowing water with her hands and feet. But she cannot use her feet in this grouped position (S=5). One of them slips towards a dorsal support (S=5), the other moves away. There is a torsion around the longitudinal axis (S=4).

Sitting in line with the bathtub, she tries to move her arms back and forth to make waves. Her sitting position in the water is not balanced. She tilts in all directions like a culbuto and has to use her hands to restore her sitting position (S=4). From the 14th month her sitting position becomes stabilized. Sometimes, an upper limb is placed behind the trunk in search of support (S=2) (Fig. 3I-J). She brings it forward when asked. She cannot yet rest the soles of her feet on the wall. Any change in position or emotion causes her body to twist (S=4) (Fig. 3K). When she is pushed back and forth in a sitting position, her lower limbs are stiff, her supinated feet (S=5) rest on the outer edges and her hands rest on either side. At 15th month, when Bahiya is placed in the supine position, she is stiff (S=4). Her body is twisted with a slight flexion of the hips (Fig. 3L). She has to be held under each shoulder; she quickly straightens up, using her hands for support. However, she accepts being rocked back and forth with her feet on the wall to restart the return movement. This restart is weak because her feet are in supination and her legs remain stiff. During the return movement, she moves to the sitting position.

In Period V, the child is offered a position that is grouped across the width of the tub. In this rolling position we work on the effect of sole support on verticality and distal involvement. When the pelvis slides toward the heels in this position, the sole support decreases (S=3). The therapist therefore suggests that Bahiya find a way to stand up without using her upper limbs and by helping her to keep her knees together. The sole support is reintroduced and the sitting

position is made comfortable. She then progresses in her sensory experiences with water. Sitting along the length of the tub, Bahiya's lower limbs rise to the surface (S=4) as she handles the bottle (Fig. 3M, N). By helping her push down on the bottle, we tilt her center of gravity forward. By repeating this experience over several sessions, she becomes able to rebalance her body in the horizontal and sagittal planes, which improves the involvement of her upper limbs and the use of her thumbs in grasping (S=1). Relieved by the support of her feet, she lies in the decubitus position (S=2); her lower limbs are stiff, her feet in supination. During the passive movements to and fro, Bahiya's body gradually turns to the right (Fig. 3O) (S=3). Supported under the first dorsal vertebrae, Bahiya remains supine but stiff. She has to be helped to bend her lower limbs so that she can feel the possible sole support on the bottom of the tub. However, to maintain her posture, she does not use a sole support, but a support with the dorsal side of her feet under the therapist's hands (S=3). Her lower limbs quickly contract in extension. At 20th month, she is sometimes able to voluntarily find support at the bottom of the bathtub by bending her knees. At the end of period V, she lies down on her own, allowing the water to reach her mouth and ears (S=1). She likes to slide in, letting her feet push and pull her. Her body still bends to the right when she pushes (S=5).

In the last period, Bahiya now sits on both buttocks to play with the jet. In the sitting position we can see a progression in the support of the feet on the walls, which often starts with the dorsal side of the foot and then evolves to support on the toes (S=1). When lying down, a little help is enough to keep the feet on the bottom of the bathtub and the body afloat. Supported in the decubitus position, Bahiya manages to float. The tonic variation of her lower limbs, alternately stiff and relaxed, as she pushes the end of the bathtub with her feet, shows that the representation of her lower limbs is not yet complete and that the required voluntary action is complex for her. Alone, Bahiya lies down and stabilizes her body by supporting herself

laterally with the outer edges of both feet. In this position, she plays with dipping her mouth and ears or experiments with different water games (S=2). However, the hands soon regain their balance and lose their exploratory function (S=1).
At the end of this period, she can flap her legs in vertical movements. In procubitus, she likes to splash by moving her legs. However, the pelvis twists and the whole upper body remains passive in a supporting role, which we try to avoid.

Gradually, the support of the soles of her feet on the hands of the psychomotricist and the support of her head on the sloping wall of the tub allow Bahiya to float. This sole support can be used to initiate gentle rocking movements. Supination tends to fade for longer and longer periods of time. The curvature of the trunk follows the same progression (S = 1). Sitting up, she can now spontaneously find tonic recruitment of the lower limbs (S=2) and support on the bottom of the bathtub (S=2), thus freeing her hands (S=1).

Fine motor coordination and adaptation to objects

In Period I, Bahiya empties and fills bags with small objects and mixes building blocks in a multi-sensory search. She plays with modeling clay for long periods of time. Some objects are held to her mouth. She can do insert puzzles with geometric shapes with a little help. She also has imitation skills, which can be seen in the dinner game with the doll. In terms of graphics, Bahiya scribbles without paying attention to demonstrations. In Period II, Bahiya understands that she needs to sit in front of the table to access fine motor activities. As she sits on the edge of the chair, her knees remain bent to the left and her feet rest on her curled toes (Fig.3P-R). By adjusting the footrests with a flexible tactile pad under the feet, the seat can be repositioned at the back of the chair. Correction of the lower limb position is required at each

session. Bahiya enjoys insert puzzles; she matches shapes and colors (S=3). The speed and accuracy of her responses correlate with the symmetrical balance of her posture. Simple manipulations of modeling clay become possible without her immediately putting it in her mouth. In terms of graphics, the doodles become denser and more centered on the page. After the demonstration, the beginnings of coloring appear (S=3).

In period III, Bahiya makes choices about activities and respects the framework of the sessions, maintaining the relationship with the therapist. Puzzles become more complex. While two-handed coordination is being established, a little help with grasping is needed to hold a small fishing rod with a magnetic line. Her success is enhanced by keeping her feet on the sensory floor tile, although her posture is spontaneously quite well balanced. The use of an uneven tile makes it easier to keep her feet flat and helps Bahiya better distinguish the shapes of the puzzle pieces and fit them together. Bahiya's pencil grip is still immature. She explores her graphic space by drawing an outline of the page and then tries to erase the overhangs on the table (S=3). However, the use of the thumb remains low (S=4).

Distal disinvolvement can be observed in period IV in the bath. To support the shower head, she has to rest it and her forearm on her thigh (Fig. 3S) (S=5). In the transverse position in the bathtub, she is unable to throw water against the wall in front of her. Her upper limbs move back and forth, but her hands remain inactive in flexion (Fig. 3T, U) (S=4). Similarly, when she makes waves, her hands and fingers offer no resistance to the water and the gesture remains ineffective. Sitting opposite the faucet, with her back to one wall and her feet to the other, Bahiya is invited to explore the flowing water with her hands and feet. She tries to catch the "water stick" a few times. Gradually the support of the feet on the opposite wall is extended. Then the two-handed coordination is organized to make a bowl under the water jet or to play

with the water pressure (S=2). When the bathtub empties, Bahiya is visually attracted by the suction of the water in the drain. With her body twisted, she puts her hand in to feel the suction under her fingers. Finally, she tries to drink the whirlpool (S=3). In period V, as Bahiya progresses in the organization of her sitting posture in front of the water jet, sensory experiences develop together; for example, she tries to grasp between her thumb and index finger the thin film of water cascading from the adult's hand (S=1). At the beginning of this period, Bahiya is unable to push an empty plastic bottle into the water. She has to be helped to place her hands on either side of the bottle, with her thumbs opposite her other fingers (S=1). But Bahiya quickly removes her thumbs from the grip. She still needs help to hold the filled bottle above the surface to empty it. She is only able to hold the bottle when her forearms are supported or when she presses it against her thighs (S=1), but her thumbs are not involved. After a few sessions, Bahiya is able to hold the bottle underwater with her hands and thumbs in opposition, then with one hand. She can now hold the bottle above the surface to empty it without resting her forearms on her thighs (S=1). At the end of period V, she initiates new watering games. During period VI, Bahiya lies in the water and experiments with moving the water with her hands or using the shower head to spray water in different ways. The open hands can exert a force on the water and tap its surface (S=2). Sometimes, in a fit of excitement, Bahiya throws the water around and makes waves (S=2). She pauses to watch the splashes outside the tub, demonstrating the awareness she has developed of the effect of her actions on the environment. During these rapid movements, the fingers may lose their tone.

At the beginning of the study, Bahiya has little involvement in what we call the relational grip (S=4). In period II, Bahiya holds the adult's hands with all four fingers to pull the ball (S=3). In periods IV and V, this grasp develops considerably with work in the water (S=1). In period VI,

she voluntarily gives her hand to come to the session (S=1). She grasps the adult's hands to support herself when jumping on the trampoline (S=1).

Communication and language

At the end of the first period, Bahiya begins to grasp the picture offered to her and to return it upon gestural and verbal request (S=4). She responds to very few simple instructions and does not turn around when her first name is called (S=4). Bahiya does not use pointing, does not make requests, picks up toys of interest on her own, produces syllables and occasionally says a few words. Expressive language and nonverbal communication are rated at 5. In Period II, vocalizations are still often associated with a rictus (S=4), but they accompany her requests, handling of objects, related play, and emotional expression. The exchange of pictures increases (S=3). The parents notice a contextual increase in the lexical inventory, which we do not observe in the sessions. Despite the development of eye contact (S=4) and smiling (S=3), nonverbal communication remains poor (S=4). In period III, Bahiya participates better in the exchange of pictures (S=3). Proto-imperative pointing is observed at home. She tends to express her wishes by looking and reaching for what she wants. In order to repeat a song, she can take the adult's hands and make him sketch the first gesture of the rhyme. Eye contact is now frequent and used to get help (S=3). Nonverbal communication is therefore improving (S=3). In Period IV, when named by pointing, Bahiya leans forward until the adult's finger touches her. Some imitations appear in water games (S=2). She shows interest in swimming by repeating the little rhyme that accompanies it. Proto-declarative pointing appears. In period V, some new words appear and she begins to sing in English (S=3). When asked, Bahiya can fetch the bath picture and give it to the adult (S=2). She discovers her navel, points to it,

and introduces it in a monosyllable while looking at the adult. In Period VI, pictures support Bahiya's communication at school. In the unit, she sometimes calls out to the children with her voice and eyes. She participates orally in a song by repeating "hand, leg, foot, head" (S=3). Her pronunciation is very approximate. She uses the "goodbye" and "put away" gestures appropriately and can combine several gestures to communicate and express emotions. For example, she looks mischievously at the adult and uses the "cry" gesture to indicate that she does not like what is being offered.

Sociability and behavior

At the beginning of Period I, eye contact is infrequent and consists of a sidelong glance accompanied by a rictus. She does not play with other children and participates very little in school activities. Bahiya is calm when sitting on her mother's lap with her back pressed against her, but when standing, she often bangs her forehead or chin against her mother's arm. To express displeasure, she bangs her forehead on hard surfaces and uses the digitigrade gait or jumps. When sitting on a small chair, she often tips backwards and sometimes falls (S=5). However, she was soon happy to come to the session. Self-aggressive episodes decreased significantly during the first months. Bahiya participates in dressing; she has achieved daytime sphincter continence. She eats on her own, but awkwardly. She has no special dietary requirements. In period II her progress is noticed by her parents. She is more in touch with her environment (S=3). Her tantrums persist when she is frustrated. Although she is independent in certain daily activities, such as putting on her coat, her play is still poor. All the actions that Bahiya masters are done in a hurry. In period III, despite progress in the relationship, Bahiya remains restless at home (S=3). She continues to have difficulty sleeping, eating, and accepting

restrictions on screen exposure. She now agrees to have her hair done. Her adaptation to the school environment is progressing. Activities (balloon exchange, dinette, etc.) remain repetitive and stereotyped. However, she shows enthusiasm when her body axes are challenged (squatting, trampoline, bodyball).

From Period IV on, working in the water stimulates Bahiya's senses and she shows interest in these new experiences. In period V, Bahiya is more sociable, but isolates herself during playtime. Accompanied by an adult, she remains attentive to school activities and no longer throws objects (S=3). In the unit, while waiting for her session, she sits around a small table with other children (S=2) whom she observes and engages in various fine motor activities. She is becoming almost completely independent in her dressing. In the last session, Bahiya gets up as soon as the therapist comes to pick her up, gives her a few looks, and puts away the activities in progress (S=1). On the way she may call to another child (S=1).

Correlation between data

Figure 4B shows the evolution of Bahiya's scores in the different domains. As observed with Saral, there is a progressive decrease in the scores for all the items and a strong correlation between the scores obtained in the area of postural abnormalities and in the other areas (behavior, communication, social interaction). Consistent with the diminution of the scores obtained with our observation scale was the reduction of the CARS score (39.5 at the start of the study and 30 at the end).
