# Supplementary figures and images for: Improvement of motor disorders and autistic symptomatology by an approach centered on the body axis: a two-case report
Source: Front Child Adolesc Psychiatry. 2025 Apr 14;4:1451559. doi: 10.3389/frcha.2025.1451559 (PMC12034731; doi:10.3389/frcha.2025.1451559)

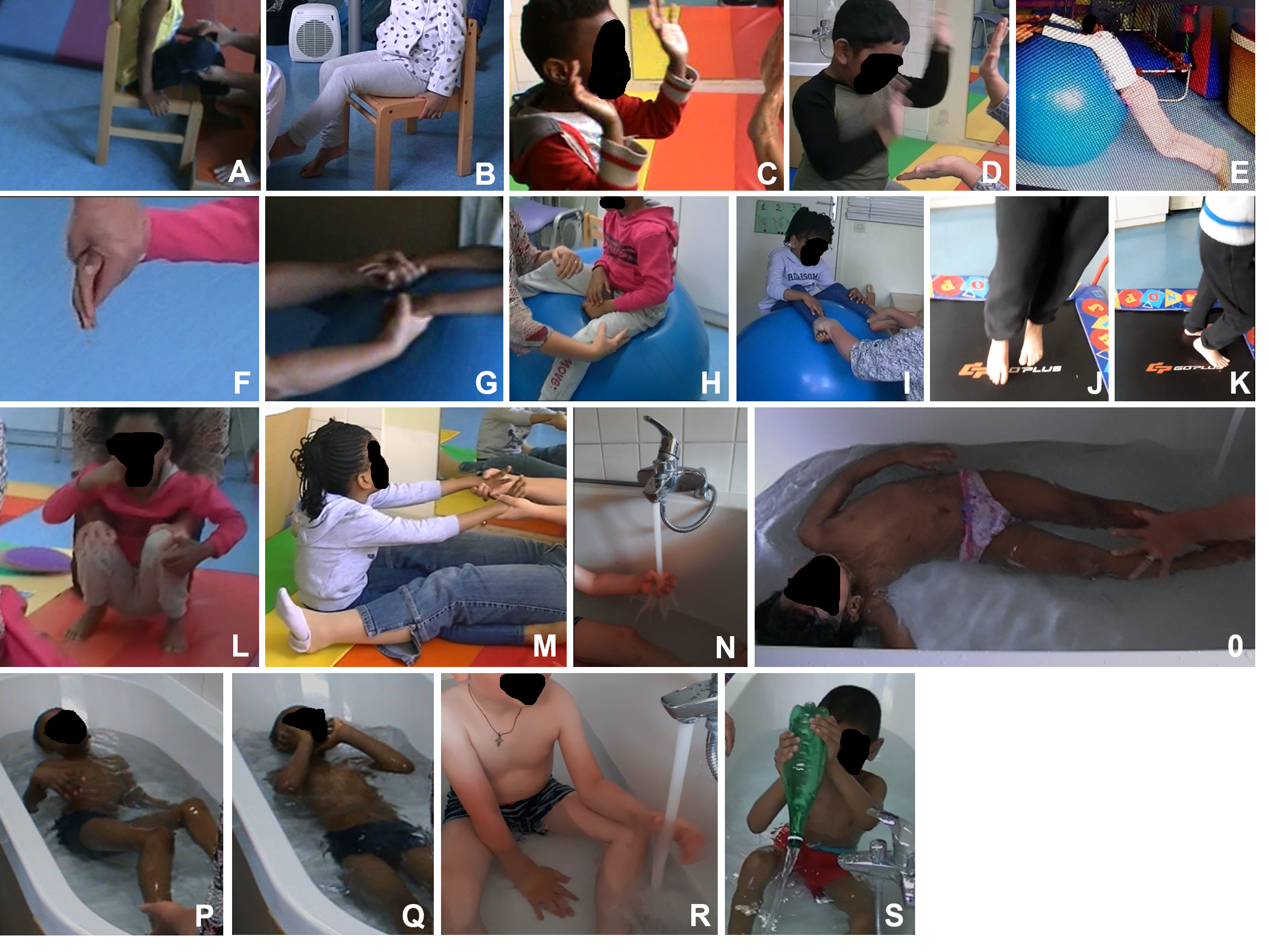

Supplement: Supplementary file 3 [file Image1.tiff]
